# Health Worker Influenza Vaccination Programs: A Key to Pandemic Preparedness and Effective COVID-19 Vaccine Deployment in Low- and Middle-Income Countries

**DOI:** 10.3390/vaccines14020130

**Published:** 2026-01-28

**Authors:** Margaret McCarron, Chelsey Griffin, Tat S Yau, Julie Garon Carlton, Jaymin C Patel, Claire B Hugo, Carsten Mantel, Lindsay B. Saber, Shoshanna Goldin, Ann Moen, Kathryn E Lafond, Jenny A Walldorf, Terri Hyde, Joseph Sewell Bresee

**Affiliations:** 1 National Center for Immunization and Respiratory Diseases, U.S. Centers for Disease Control and Prevention, Atlanta, GA 30329, USA; 2 Global Health Center, U.S. Centers for Disease Control and Prevention, Atlanta, GA 30333, USA; 3 MMGH Consulting, 8001 Zurich, Switzerland; claireb.hugo@gmail.com (C.B.H.); mantelc@mmglobalhealth.org (C.M.); 4 Task Force for Global Health, Decatur, GA 30030, USA; 5 Doctoral Program in Public Health (DrPH), Johns Hopkins University, Baltimore, MD 21205, USA

**Keywords:** pandemic preparedness, influenza, health workers, COVID-19, vaccine

## Abstract

Background/Objectives: The COVID-19 pandemic presented an urgent global need to quickly vaccinate health workers (HWs). We used this unique circumstance to assess whether mature influenza vaccination programs for HWs facilitated rapid deployment of COVID-19 vaccines in low- and middle-income countries (LMICs). Methods: We used publicly available population-level COVID-19 vaccination coverage data from the World Health Organization (WHO) COVID-19 PowerBI Dashboard for 60 LMICs to investigate general population coverage with the COVID-19 vaccine over the first year of vaccine rollout. We also evaluated country-level policy and program data reported to the WHO–United Nations Children’s Fund (UNICEF) electronic Joint Reporting Form (eJRF) in 2022 or earlier to determine if the presence of a mature (≥2 years of influenza vaccine introduction in HWs prior to 2019) HW influenza vaccination program was associated with more rapid COVID-19 vaccine deployment and/or pandemic preparedness. We used a mixed-effects beta regression model to investigate whether having a mature HW influenza vaccination program was associated with COVID-19 vaccination coverage levels and/or deployment timeliness. Finally, to provide a better understanding of the possible relationship between the presence of a mature HW influenza vaccination program and the COVID-19 vaccine rollout, we analyzed interview data collected during COVID-19 vaccine post-introduction evaluations (cPIEs). Results: Twenty-four of our study countries (40%) had mature HW influenza vaccination programs prior to the COVID-19 pandemic, and 16 (27%) participated in cPIEs. The overall adjusted mean general-population COVID-19 vaccination coverage at 12 months post-deployment in countries with mature HW influenza vaccination programs was 46% (95% CI: (35%, 56%)), compared with 25% (95% CI: (19%, 32%)) in countries without such programs. Vaccination coverage was 2.5 times higher in countries with mature programs (adjusted odds ratio 2.5, 95% CI: (1.5, 4.5); *p* = 0.001). Conclusions: Our analysis suggests that mature influenza vaccination programs for HWs were associated with timelier and more complete COVID-19 vaccination rollout.

## 1. Introduction

Vaccination is a central strategy for the prevention and control of seasonal and pandemic influenza. The World Health Organization (WHO)’s Strategic Advisory Group of Experts (SAGE) on Immunization recommends influenza vaccination for four priority groups: older adults aged 65 years and above, persons with comorbidities, pregnant women, and health workers (HWs) [1]. HWs are prioritized for their higher risk of influenza virus infection [2], their role in protecting vulnerable patients and maintaining essential health services during epidemic and pandemic periods, and for informing patients’ vaccination decisions [1,3,4,5,6,7,8,9]. Vaccinating HWs reduces absenteeism [10], thereby supporting health system resilience. Additionally, HWs were disproportionately infected with SARS-CoV-2 early in the COVID-19 pandemic [11], making them a WHO priority group for early COVID-19 vaccination [12,13].

Mature influenza vaccination programs for HWs are crucial for pandemic readiness and global health security by facilitating rapid vaccination and protecting both HWs and the public during an emergency. Seasonal influenza vaccination provides countries with an annual opportunity to exercise and improve their systems for rapid rollout of vaccines. Countries with such programs prior to the 2009 H1N1 influenza pandemic were able to procure and use vaccines more quickly than those without, due to existing infrastructure for the importation, distribution, and delivery of vaccines [14,15,16].

Despite global recommendations and evidence supporting the value of HW influenza vaccination in pandemic response, recommendations for influenza vaccination do not include HWs in most low- and middle-income countries (LMICs) [17]. Many immunization programs rely on support from Gavi, the Vaccine Alliance [18] to access routine, non-seasonal vaccines; Gavi does not currently offer support for influenza vaccines. Consequently, Africa, the Middle East, and Southeast Asia, which are home to over 50% of the global population, receive only 6% of the influenza vaccine doses distributed each year [19].

The COVID-19 pandemic provided a unique opportunity to assess whether HW influenza vaccination programs enhance pandemic preparedness. We evaluated whether countries with mature HW influenza vaccination platforms were better prepared to receive, distribute, and deliver COVID-19 vaccines and whether they achieved higher COVID vaccination coverage and faced fewer logistical challenges compared with countries without such programs.

## 2. Methods

### 2.1. Data Sources and Collection

To investigate the relationship between the HW influenza vaccination program maturity and pandemic preparedness, we evaluated country-level data for 60 LMICs reported to the WHO Immunization Data Portal (i.e., the WHO–United Nations Children’s Fund (WHO-UNICEF) electronic Joint Reporting Form or eJRF influenza module) [20] in 2022 or earlier. These data were used to determine if the presence of a mature HW influenza vaccination program, defined as ≥2 years of influenza vaccine introduction in HWs prior to 2019, was associated with COVID-19 vaccine deployment and/or pandemic preparedness. National-level data on influenza vaccination policies and vaccine availability are reported annually to the WHO by participating countries and were obtained in 2023 for these analyses. We also evaluated publicly available population-level COVID-19 vaccine immunization data from the WHO COVID-19 PowerBI Dashboard [21] and the UNICEF COVID-19 Market Dashboard [22] for differences in COVID-19 vaccination coverage over the first year of vaccine rollout. Additionally, income status was defined by the World Bank (WB) lending group (low, lower-middle, and upper-middle income) [23]. Finally, to investigate whether the number of physicians, including generalists and specialist medical practitioners, and nursing and midwifery personnel, impacted the timeliness of COVID-19 vaccine rollout and COVID-19 coverage levels, we obtained health worker density data (baseline year of 2020) from the global health security index [24] to use in our models. Inclusion in the analysis was subject to completeness of reporting to the WHO and UNICEF.

We also analyzed data from COVID-19 vaccine post-introduction evaluations (cPIEs) [25] conducted in a purposive, geographically representative sample of 16 low (1), lower-middle (11), and upper-middle income (4) countries with and without mature influenza vaccination programs for HWs. Countries were selected from those receiving funding from the U.S. Centers for Disease Control and Prevention (CDC) to support early COVID-19 vaccination programs and were interested in conducting a cPIE evaluation. The cPIEs followed standardized WHO guidance, modified from routine immunization program post-introduction assessment tools. They incorporate nine thematic areas aligned with COVID-19 National Deployment and Vaccination Plans (NDVPs): regulatory preparedness, planning and coordination, service delivery, costing and funding, supply chain and waste management, human resource management and training, vaccine demand, vaccine safety, and monitoring and evaluation. Briefly, cPIE methodology included standardized data collection tools used to gather information at the national, sub-national, and service delivery (i.e., health facility) levels via stakeholder interviews. We interviewed service delivery staff who were most involved in COVID-19 vaccination services (i.e., HWs and senior staff, including immunization program managers and clinic directors). Health facility patients receiving COVID-19 vaccines at the time of site-level data collection were also interviewed (Supplemental Table S1). Stakeholder cPIE interview questionnaires were programmed into Open Data Kit (ODK, 2023) software, and data were recorded electronically using tablets, where available. Some country teams collected survey data via paper forms and entered information into databases following collection. Survey teams in each country received 3 to 5 days of data collection training and technical support from CDC and the Partnership for International Vaccine Initiatives (PIVI) [26] staff prior to and throughout survey implementation. Where appropriate, we used a supplemental questionnaire to understand how countries with prior influenza vaccination experience leveraged components of those programs during the planning, deployment, and monitoring of COVID-19 vaccination programs. 

### 2.2. Data Analysis—Public Data

Countries were assigned income status using WB lending groups and Gavi eligibility status for 2023 using information from the organization’s website [27], and participation in PIVI was noted. Countries that reported HW influenza vaccine introduction in their 2019 eJRF report and for at least the two years prior were considered to have a mature influenza vaccination program; all others were considered to have no such program. We adopted this method to define program maturity to facilitate comparability of findings with other analyses [28]. An initial definition of program maturity was made via consultation with influenza vaccination program experts, based on the quality and availability of influenza vaccination data elements reported by LMICs to eJRF, and is described by Goldin et al. [28]. We selected ≥2 years prior to 2019 as the threshold for program maturity to ensure that countries had several seasons of experience with influenza vaccines in the targeted population. 

We calculated overall immunization program maturity scores (one each for influenza and for diphtheria-tetanus-pertussis (DTP)/Pentavalent) and COVID-19 capacity scores (in 2020) for all countries included in the analyses, using previously published formulas [28] (Supplemental Tables S2 and S3); these variables were included due to their potential to confound our findings. The influenza and DTP immunization program maturity scores assessed vaccination coverage, policy, and vaccine introduction data from the past 20 years and were used as a proxy for measuring the maturity level of each country’s national immunization program (NIP). The DTP/Pentavalent NIP maturity score range was 0 through 5, with a score of 5 representing a fully mature NIP. The influenza NIP maturity score range was 0 through 4, with a score of 4 representing a fully mature influenza vaccination program. The maturity level of a country is likely to differ according to the number of years of program experience. The COVID-19 capacity score assessed each country’s capacity for evidence-based decision-making, waste management, vaccine safety surveillance, disease surveillance, and vaccine acceptance and demand during the COVID-19 vaccine rollout. COVID-19 capacity scores ranged from 0 to 5, with a score of 5 representing high capacity to deploy COVID-19 vaccines during the pandemic.

To understand differences in the speed of COVID-19 vaccine rollout associated with the presence or absence of mature HW influenza vaccination programs, we compared the monthly proportion of countries in each group that had begun administering COVID-19 vaccination in the general population over the first year of vaccine availability. To address differing vaccine delivery dates across countries, our analysis was adjusted for the first day of the first month of vaccine deployment in each country.

To identify potential associations between COVID-19 vaccination coverage in the general population and HW influenza vaccination program maturity, we compared COVID-19 vaccination coverage (i.e., the number of doses administered per 100 persons) for the vaccine-eligible general population during the first year following the deployment of COVID-19 vaccines. COVID-19 vaccination coverage data were analyzed at intervals (1, 3, 6, 9, and 12 months); month 0 represented the first month of vaccination. We implemented a mixed-effects beta regression model with a random intercept for country to account for repeated measures and unobserved between-country differences. Vaccination coverage was expressed as a proportion on the 0 to 1 scale. Vaccination coverage in countries with and without mature influenza vaccination programs for HWs was compared using the emmeans package in R [29] to estimate odds ratios with 95% confidence intervals. We investigated potential confounding factors by assessing the impact of the following on COVID-19 vaccination coverage: country HW density, participation in PIVI, 2019 gross domestic product (GDP) per capita, NIP, influenza vaccination program maturity scores, and COVID-19 capacity scores. We further stratified countries with and without mature HW influenza vaccination programs by WB lending groups to explore differences among countries with similar incomes. 

### 2.3. Data Analysis—cPIE Data

We analyzed interview data collected using the cPIE tools to investigate commonalities among key stakeholders in countries with and without mature influenza vaccination programs for HWs. Country cPIE datasets were cleaned and merged into single pooled global databases per survey administrative level. We measured planning and preparedness at the national level by comparing the number of countries that reported having previously existing influenza pandemic preparedness plans and whether those plans were adapted for the development of COVID-19 NDVPs in each country group. We also quantified the reported presence of established regulatory pathways for new vaccines by investigating regulatory barriers to vaccine rollout. Finally, we measured the reported re-use of existing tools and infrastructure established by the national immunization program or the influenza vaccination program (e.g., microplanning tools, vaccine storage, and cold chain status).

At the health facility level, supply chain management, vaccine demand generation, and recording and reporting practices were investigated. We also measured the reported reuse and repurposing of existing tools for COVID-19 vaccination services. Differences in HWs’ responses related to their COVID-19 vaccine acceptance were investigated and compared across different groups—influenza program maturity, geographical regions, and income levels. 

Statistical analyses were performed using both Microsoft Excel and R (version 4.4.1) software.

### 2.4. Partners and Ethical Review

Organizations that supported and implemented this work from 2021 to 2024 included the CDC and funded partners, including the Task Force for Global Health (TFGH), specifically PIVI, national ministries of health, and MMGH Consulting, as well as unfunded technical collaborators at the WHO. Ethical review was conducted by the CDC, deemed not research, and was conducted consistent with applicable federal law and CDC policy (see e.g., 45 C.F.R. part 46; 21 C.F.R. part 56; 42 U.S.C. §241 (d); 5 U.S.C. §552a; and 44 U.S.C. §3501 et seq.). No personal data on human subjects was collected.

## 3. Results

### 3.1. Study Population (Public Data)

We included 60 low- and middle-income countries in our analysis, representing all WHO regions—African (24, 40%), European (12, 20%), American (9, 15%), Western Pacific (6, 10%), Southeast Asia (5, 8%), and Eastern Mediterranean (4, 7%). Twenty-four countries (40%) had mature HW influenza vaccination programs for at least 2 years prior to the COVID-19 pandemic. The 60 countries were in the low (14, 23%), lower-middle (28, 47%), and upper-middle (18, 30%) income World Bank lending groups. The median GDP per capita for mature HW influenza vaccination program countries and non-mature HW influenza vaccination program countries in 2019 was USD 4555 (range: 1959–12,885) and USD 1390 (range: 500–8906), respectively. Twenty-nine (48%) countries from this sample were eligible for Gavi support in 2023, 16 (27%) were PIVI partners, and 16 (27%) participated in CDC-PIVI-supported cPIEs (Table 1). National immunization program (NIP) maturity levels varied across countries included in this analysis; NIP maturity scores were typically higher among countries with a mature HW influenza vaccination program than those without such programs (Table 2).

### 3.2. Timely Deployment of COVID-19 Vaccines in Country (Public Data—60 Countries)

**Figure 1:**
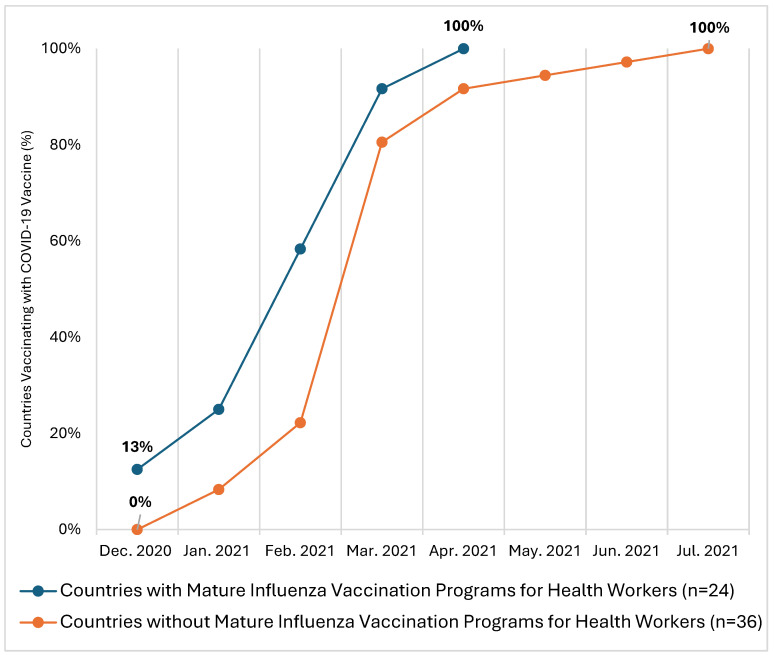
COVID-19 vaccine deployment by month, by presence of a mature influenza vaccination program; publicly available data (*n* = 60 countries).

The first COVID-19 vaccines were available for global delivery in December 2020. Based on WHO-UNICEF data, 25% of countries (6 of 24) with mature influenza vaccination programs for HWs began COVID-19 vaccination in the general population by January 2021, and all 24 were vaccinating by April 2021, 4 months into the global rollout of COVID-19 vaccines. In comparison, 8% of countries (3 of 36) without mature influenza vaccination programs for HWs started COVID-19 vaccination by January 2021, 92% of those countries (33 of 36) were vaccinating by April 2021, and all were vaccinating by July 2021 (Figure 1). When stratified by income group, a higher percentage of upper-middle-income countries with mature HW influenza vaccination programs (*n* = 2, 13%) began COVID-19 vaccination earlier (December 2020) than upper-middle-income countries without such a program (*n* = 0, 0%) (Supplemental Figure S1).

### 3.3. COVID-19 General Population Vaccination Coverage (Public Data—60 Countries)

**Figure 2:**
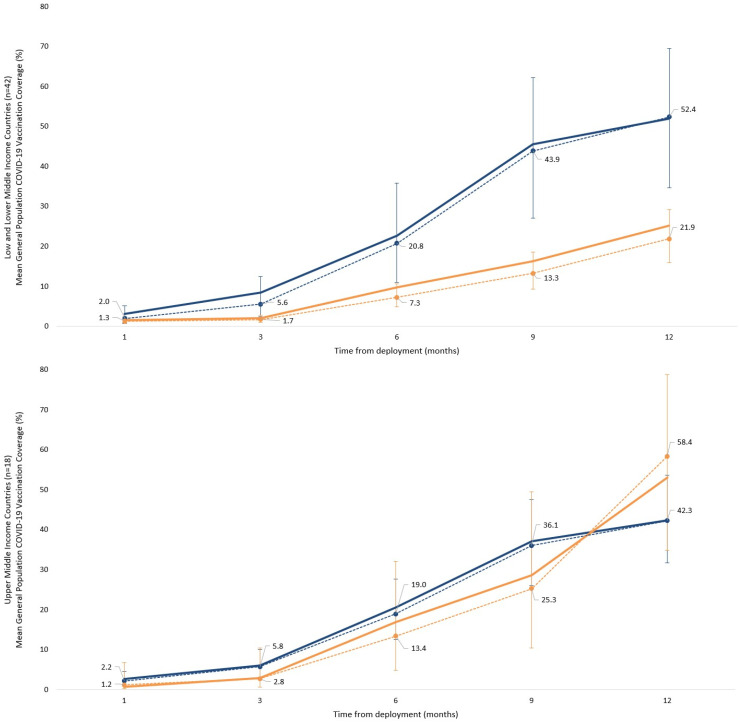
General population COVID-19 vaccination coverage 12 months following COVID-19 vaccine deployment, by mature HW influenza vaccination program presence and World Bank income classification; publicly available data (n = 60 countries). Solid lines show mean coverage; dashed lines show country-adjusted model estimates with 95% confidence intervals. Blue lines refer to countries with mature HW influenza vaccination program; orange lines indicate countries with no such mature program.

We found that at 12 months post-deployment, model-based adjusted mean general population COVID-19 vaccination coverage from the mixed-effects beta regression (adjusted for timepoint and a country-level random intercept) was 46% (95% CI: (35%, 56%)) in countries with mature HW influenza vaccination programs versus 25% (95% CI: (19%, 32%)) in countries without. The coverage was 2.5 times higher in countries with mature HW influenza vaccination programs (adjusted odds ratio of 2.5, 95% CI: (1.5, 4.5); *p* = 0.001) (Supplemental Table S4). Among low- and lower-middle-income countries, 12 months after deployment, a mixed-effects beta regression estimated an adjusted mean coverage of 52% (95% CI: (35%, 70%)) for countries with mature health worker influenza vaccination programs, compared with 22% (95% CI: (16%, 29%)) for those without. COVID-19 general population vaccination coverages were higher with mature HW influenza vaccination programs (adjusted odds ratio of 3.9, 95% CI: (1.7, 9.1); *p* = 0.001). In upper-middle-income countries, no statistically significant difference was observed at 12 months (Figure 2, Supplemental Table S4).

To assess confounding/effect modification, we evaluated the interaction between the mature influenza vaccination program and geographic regions and the interaction between the program and the World Bank lending groups. The joint tests for interaction were not statistically significant. Given sparse cells in some strata, the power for evaluating interaction terms was limited.

### 3.4. cPIE Findings

In addition to the publicly available data, 16 countries contributed in-depth interview data from a cPIE, including 7 (44%) with mature influenza vaccination programs for HWs for at least 2 years prior to the COVID-19 pandemic (Table 1). At the national level, 5 (71%) countries with mature influenza vaccination programs for HWs reported using their special service influenza vaccine delivery platforms, such as mobile (*n* = 2) or workplace vaccination (*n* = 3), for new COVID-19 vaccination services. Of the five countries with a mature influenza vaccination program for HWs that responded to the question, most (*n* = 4) reported adapting their influenza pandemic preparedness plans for COVID-19, including repurposing their national pandemic influenza (vaccine) deployment and vaccination plans. From the eleven responding countries, only one of five countries with a mature influenza vaccination program for HWs reported regulatory barriers or delays in obtaining required approvals or import permits for COVID-19 vaccines, while two countries that do not have a mature influenza vaccination program for HWs (N = 6) reported experiencing such barriers (Supplementary Materials).

At the health facility level, 5% (*n* = 3) of 65 senior staff interviewed in countries with mature influenza vaccination programs for HWs reported inadequate cold chain capacity for COVID-19 vaccines, compared with 24% (13 of 54) in countries without mature influenza vaccination programs for HWs (*p* = 0.002). Commonly reported problems with the supply chain included insufficient vaccine carrier supply to maintain the cold chain (i.e., cold boxes or freezer packs) and faulty and/or low-capacity cold or ultra-cold storage during vaccine rollout. One third (37%, 28 of 76) of responding health facility senior staff reported use of a vaccine reporting system already in use for other vaccines. A higher proportion of countries without mature influenza vaccination programs (22 of 45, (49%) repurposed immunization tools used for influenza, compared to countries with mature influenza vaccination programs for HWs (6 of 31 (19%); *p* = 0.009) (Supplementary Materials).

Most senior health facility staff reported using multiple modalities for demand generation to reach target groups and said they relied on the same communication channels and strategies as those used to generate demand for influenza vaccines. These included mass media/broadcast messaging (e.g., television, radio, and newspapers), social media messaging, and engaging trusted messengers (e.g., community and religious leaders) to share information about the benefits of vaccination. When asked how they leveraged the influenza vaccination program for delivery of COVID-19 vaccines, immunization managers and clinic directors among both country groups said they used existing relationships and registries of persons in groups at higher risk for complications from influenza to reach the same populations for COVID-19 vaccination (Supplementary Materials).

Overall, most (99%, 256 of 262) HWs interviewed during cPIEs reported receiving the COVID-19 vaccine within the WHO-recommended time frame [13]. Notably, 92% (*n* = 59) of HWs from countries with mature influenza vaccination programs for HWs who participated in cPIE interviews reported receiving booster doses compared to 69% (*n* = 78) of the HWs in countries without mature influenza vaccination programs for HWs at the time of participation in the cPIE evaluation. Booster dose acceptance among surveyed HWs was associated with income level, particularly within the lower-middle-income group (*p* = 0.011) (Supplemental Table S5).

## 4. Discussion

Our analysis of COVID-19 vaccine rollout in LMICs and key stakeholder interviews illustrates how the presence of a mature HW influenza vaccination program was associated with more successful vaccine deployment and higher COVID-19 vaccination coverage among both the general population and HWs. This association persisted even after accounting for important potential confounders, such as DTP and influenza national immunization program maturity and country GDP. Although we accounted for immunization program maturity and country income, there may be additional unmeasured factors that would facilitate receipt of outside support, such as external funding for immunization programs, that we were not able to fully measure.

The findings contribute to broader evidence that having mature influenza vaccination programs is a key element of pandemic preparedness and response [28,30,31]. Countries with mature immunization programs experienced greater successes and fewer setbacks during the COVID-19 vaccine rollout. Quicker COVID-19 vaccine deployment and higher vaccination coverage suggest that existing, mature immunization infrastructures, like the polio infrastructure in the African region, were effectively used for COVID-19 vaccine distribution [32], further demonstrated by the countries participating in cPIEs. The benefits of mature influenza vaccination programs for HWs extended to multiple aspects of COVID-19 vaccine rollout, from coverage to logistics and planning, and vaccine demand. 

Countries with mature influenza vaccination programs for HWs received COVID-19 vaccines earlier and were able to begin delivering doses more quickly. The differences we observed in COVID-19 vaccine distribution activities in LMICs also mirrored national experiences during the 2009 influenza pandemic, observed by Porter et al. [15]. Low-income countries generally began vaccinating later, and African countries, which had the fewest mature HW influenza vaccination programs, faced significant delays. Similarly, in 2009–10, African countries received 12 million doses of vaccine but administered only two-thirds due to regulatory obstacles, shipping delays, fractured deployment plans, low vaccine acceptance, and safety concerns [14]. During the same period, Latin American countries with seasonal influenza programs received pandemic influenza vaccine earlier than those without such programs during the 2009 influenza pandemic [16]. COVID-19 vaccine access and availability differed for all countries, and in some cases, countries that were ready to receive and deploy vaccines were unable to procure them due to various challenges, including stockpiling by more developed countries and limited donations to LMICs [33]. Variability in access to COVID-19 vaccines also resulted in delays in achieving target coverage [34].

Our findings align with other analyses from the COVID-19 pandemic period, which identified certain national characteristics (the presence of childhood, adolescent, and adult vaccination programs; higher income status; high health workforce density; and high population trust in government leaders and health workers) that contribute to COVID-19 vaccination program success. Notably, an effort led by Goldin et al. [28] showed that among these characteristics, having a seasonal influenza vaccination program was significantly associated with both higher COVID-19 vaccination coverage rates and stronger capacities to implement COVID-19 vaccination.

Likely due to country mandates, there was no significant difference in reported primary series vaccination among HWs in countries with and without mature influenza vaccination programs for HWs. However, HWs in countries with mature HW influenza vaccination programs who participated in cPIEs reported higher COVID-19 vaccination booster dose uptake, consistent with findings that previous acceptance of influenza vaccines was associated with higher COVID-19 vaccination rates and continued receipt of vaccination [35,36]; Jorgensen et al. found that HWs in Albania previously vaccinated against influenza were twice as likely to receive COVID-19 booster doses [37]. The cPIE findings demonstrate more frequent recommendations for both influenza and COVID-19 vaccines to patients in countries with mature HW influenza vaccination programs. These findings are aligned with previous studies that have also shown that prior vaccination among HWs against influenza was a strong predictor of acceptance and recommendation of other vaccines, including the COVID-19 vaccine [7,38].

It is important to recognize that the rapid mobilization of COVID-19 vaccination programs was propelled by a global crisis and related political will. While it is true that crisis-driven political mobilization may account for some degree of rapidity in the successful rollout of COVID-19 vaccines globally, we are unable to measure the impact of such forces. Our conclusions are consistent with those of other researchers in their demonstration of the role of well-established, mature vaccination programs in successful COVID-19 vaccine rollout and pandemic preparedness.

## 5. Limitations

Our analysis is subject to some limitations. First, cPIE data were collected by different teams across 16 different countries with varying levels of data collection training. In some cases, cPIE questionnaires were translated into local languages, which may have impacted interviewer/interviewee understanding of questions; in some cases, this resulted in data gaps due to incomplete questionnaires. While every effort was made to collect missing data, not all data could be recovered. In such cases, those data points were omitted from calculations. Second, the timing of cPIE implementation varied considerably by country, and responses may have been subject to recall bias. The cPIEs were conducted from 2021 to 2023, during which countries were at different stages of COVID-19 vaccination program implementation. Nevertheless, we believe the cPIE data presented are sufficiently representative to report. Third, COVID-19 vaccination reporting was not consistent across all countries. We excluded incomplete data whenever possible; some point estimates might be biased by missing data. Finally, we did not have complete data on other HW vaccinations (e.g., hepatitis B, measles, and yellow fever), so no analysis was performed to determine if these programs impacted COVID-19 vaccine deployment, coverage, or acceptance. However, we were able to explore the impact of other non-HW vaccination programs (e.g., DTP) to account for broader themes in immunization system strength.

## 6. Conclusions

These data underscore the role of mature seasonal HW influenza vaccination programs in support of effective and timely vaccination during a pandemic, enabling a country to effectively implement its pandemic vaccination program and reach targeted populations more quickly. High COVID-19 vaccination coverage among HWs in LMICs with mature HW influenza vaccination programs reaffirms this, as most HWs receive an annual seasonal influenza vaccine. Significant differences in both COVID-19 vaccination coverage and the speed of vaccine deployment highlight how mature immunization frameworks for key adult populations, such as HWs, can bolster pandemic preparedness and ensure swift and successful responses to future public health emergencies. Maintaining and strengthening critical immunization structures in interpandemic periods may help to protect both HWs and the general population globally against new and emerging infectious disease threats.

## Figures and Tables

**Table 1:** Description of data sources, including the presence or absence of a mature health worker influenza vaccination program.

Public Data Countries (*n* = 60)			
	Mature Health Worker Influenza Vaccination Program (*n* = 24)	No Mature Health Worker Influenza Vaccination Program (*n* = 36)	Total (*n* = 60)
**Geographic Region, *n*, (%)**			
African	0 (0)	24 (68)	24 (40)
American	9 (38)	0 (0)	9 (15)
Eastern Mediterranean	2 (8)	2 (6)	4 (7)
European	9 (38)	3 (8)	12 (20)
Southeast Asia	1 (4)	4 (11)	5 (8)
Western Pacific	3 (13)	3 (8)	6 (10)
**World Bank Income Group**			
Low Income	0 (0)	14 (39)	14 (23)
Lower-Middle Income	9 (38)	19 (53)	28 (47)
Upper-Middle Income	15 (63)	3 (8)	18 (30)
**Gavi ^#^ Eligible (per 2023)**			
Yes	1 (4)	28 (78)	29 (48)
No	23 (96)	8 (22)	31 (52)
PIVI * Partner			
Yes	9 (38)	7 (19)	16 (27)
No	15 (63)	29 (81)	44 (73)
**COVID-19 Vaccine Post-Introduction Evaluations (cPIEs) Countries (*n* = 16)**			
	**Mature Health Worker Influenza Vaccination Program** **(*n* = 7)**	**No Mature Health Worker Influenza Vaccination Program** **(*n* = 9)**	**Total** **(*n* = 16)**
**Geographic Region, *n*, (%)**			
African	0 (0)	4 (44)	4 (25)
Eastern Mediterranean	1 (14)	1 (11)	2 (13)
European	3 (43)	1 (11)	4 (25)
Southeast Asia	1 (14)	2 (22)	3 (19)
Western Pacific	2 (29)	1 (11)	3 (19)
**World Bank Income Group**			
Low Income	0 (0)	1 (11)	1 (6)
Lower-Middle Income	4 (57)	7 (78)	11 (69)
Upper-Middle Income	3 (43)	1 (11)	4 (25)
**Gavi Eligible ^#^ (per 2023)**			
Yes	0 (0)	4 (44)	4 (25)
No	7 (100)	5 (56)	12 (75)
PIVI Partner *			
Yes	6 (86)	4 (44)	10 (63)
No	1 (14)	5 (56)	6 (38)

^#^ Global Alliance for Vaccines and Immunization (Gavi, the Vaccine Alliance). * Partnership for influenza vaccine introduction.

**Table 2:** Health worker COVID-19 vaccination coverage and immunization program maturity scores by country and presence of a mature health worker influenza vaccination program for low- and middle-income countries.

				Immunization Program Maturity Scores	
Health Worker COVID-19 Vaccination Coverage	Full Series	At Least One Dose	COVID-19 Capacity Score ^1^	Influenza (Out of 4) ^2^	DTP3 (Out of 5) ^3^
Countries with Mature * Influenza Vaccination Program	p ** = 0.610	p ** = 0.787	p ** = 0.147	p ** < 0.001	p ** = 0.807
Albania	74%	85%	1	2	5
Armenia	ND ^#^	ND	1	2	5
Belize	57%	100%	5	3	4
Bolivia (Plurinational State of)	100%	100%	5	3	4
Costa Rica	100%	100%	4	2	5
El Salvador	100%	100%	3	3	5
Georgia	53%	58%	5	3	5
Guatemala	100%	100%	5	3	5
Honduras	100%	100%	4	4	5
Iraq	53%	87%	1	3	4
Jamaica	65%	75%	5	3	5
Kazakhstan	0%	0%	1	3	5
Lao People’s Democratic Republic	100%	100%	4	3	5
Mongolia	100%	100%	4	3	5
Montenegro	50%	91%	3	3	4
Nicaragua	88%	100%	3	3	5
Paraguay	67%	83%	5	3	4
Republic of Moldova	81%	85%	1	3	5
Serbia	64%	79%	1	3	3
Thailand	100%	100%	1	3	5
Tunisia	ND	ND	1	3	5
Ukraine	61%	77%	5	2	4
Uzbekistan	83%	100%	1	4	5
Viet Nam	100%	100%	2	2	3
*Countries without Mature Influenza Vaccination Program*					
Bhutan	93%	100%	4	2	5
Burkina Faso	78%	82%	4	0	5
Cambodia	97%	100%	5	0	5
Côte d’Ivoire	39%	67%	5	2	5
Democratic Republic of the Congo	100%	100%	1	0	4
Eswatini	100%	100%	3	0	5
Ethiopia	56%	77%	2	0	5
Ghana	66%	78%	4	1	5
Indonesia	100%	100%	4	2	4
Kenya	53%	54%	2	2	5
Kosovo	ND	ND	1	2	2
Kyrgyzstan	59%	67%	4	2	5
Lebanon			0	0	5
Lesotho	24%	25%	1	0	5
Liberia	42%	42%	1	0	5
Madagascar	55%	61%	2	0	5
Malawi	100%	100%	0	0	5
Mali	100%	100%	1	0	4
Mozambique	49%	63%	4	0	5
Namibia	79%	97%	0	2	5
Nepal	100%	100%	4	0	5
Nigeria	54%	78%	2	0	4
Pakistan	100%	100%	5	0	4
Papua New Guinea	100%	100%	3	0	3
Philippines	100%	100%	3	3	4
Rwanda	100%	100%	0	0	5
Senegal	100%	100%	3	1	3
Sierra Leone	100%	100%	1	0	5
South Sudan	100%	100%	1	0	2
Sri Lanka	100%	100%	3	0	3
Tajikistan	91%	94%	4	1	3
Togo	70%	74%	1	1	5
Uganda	100%	100%	1	0	5
United Republic of Tanzania	ND	ND	1	0	5
Zambia	100%	100%	3	0	5
Zimbabwe	ND	ND	1	0	5

* Influenza vaccination program maturity was based on country eJRF reporting of health worker influenza vaccine introduction in 2019 and for at least 2 years prior; ^#^ ND—No data provided. ** Wilcoxon rank-sum test *p*-values between countries with and without the mature influenza vaccination program for health workers. ^1^ A score of 5 represents the highest COVID-19 capacity score [28]. ^2^ A score of 4 represents the highest influenza immunization program maturity score [28]. ^3^ A score of 5 represents the highest DTP3 immunization program maturity score [28].

## Data Availability

Data are owned by each country and can be requested.

## Supplementary Materials

The following supporting information can be downloaded at https://www.mdpi.com/article/10.3390/vaccines14020130/s1, Table S1: Health Facility Characteristics, cPIE countries, by WHO region; Table S2. National Immunization Program Maturity Scoring Rubric; Table S3. COVID-19 Capacity Scoring Rubric; Figure S1. COVID-19 vaccine rollout by month, by mature HW influenza vaccination program presence and World Bank income classification; publicly available data (n = 60 countries); Table S4. General population COVID-19 vaccination coverage 12 months following COVID-19 vaccine deployment, by mature HW influenza vaccination program presence, adjusted for country; publicly available data (n = 60 countries); Table S5. Reported health worker COVID-19 vaccination coverage, cPIE responses, by mature HW influenza vaccination program presence; (n = 10 countries; 262 health workers=).
