# Supplementary material for: Health Worker Influenza Vaccination Programs: A Key to Pandemic Preparedness and Effective COVID-19 Vaccine Deployment in Low- and Middle-Income Countries
Source: Vaccines (Basel). 2026 Jan 28;14(2):130. doi: 10.3390/vaccines14020130 (PMC12945195; doi:10.3390/vaccines14020130)
Supplement: Supplementary file 1 [file vaccines-14-00130-s001.zip › vaccines-4064085-supplementary.pdf]

Supplementary Materials

Table S1: Health Facility Characteristics, cPIE countries, by WHO region

Geographic Region *	Country	Location	Facility Type(s)	Facility Level(s)	Dates of Data Collection	Interviewees+	Sector
AFR	Cote d'Ivoire	Urban, rural	Health facilities, workplace, other&	District level, health center, local level health centers, tertiary level health center	April 2022	Health Facility Directors: 4 Health Workers: 14 Patients: 7	Public
	Ghana	Urban, peri-urban, rural	Health facilities	ND#	March 2021	Health Facility Directors: ND Health Workers: 72 Patients: 72	ND
	Kenya	Urban, rural	ND	ND	March 2021	ND	ND
	Mali	Urban, rural	Health facilities	Local/primary level health centers	March 2021	Health Facility Directors: 13 Health Workers: 16 Patients: 13	Public, private
EMR	Lebanon	Urban, peri-urban, rural	Health facilities	District level, hospitals, local level health centers, primary level health centers	March 2021	Health Facility Directors: 16 Health Workers: 27 Patients: 78	Public, private
	Tunisia^	Urban, peri-urban, rural	Health facilities, other	Local/primary level health centers, secondary and tertiary health centers, other	February 2022	Health Facility Directors: 18 Health Workers: 24 Patients: 24	Public
EUR	Albania^	Urban, rural	Health facilities	District level health centers, other	March 2021	Health Facility Directors: 8 Health Workers: 11 Patients: 6	Public
	Armenia^	Urban, peri-urban, rural	Health facilities	Ambulatory, healthcare center, hospital, polyclinics, local level health centers, medical center, primary level health centers	March 2021	Health Facility Directors: 7 Health Workers: 15 Patients: 16	Public
	Tajikistan	Urban, peri-urban, rural	Health facilities	ND	April 2023	Health Facility Directors: 20 Health Workers: 24 Patients: 10	Public
	Uzbekistan^	ND	ND	ND	March 2021	ND	ND

	Indonesia	ND	ND	ND	September 2022	ND	ND
SEAR	Nepal	Urban, peri-urban, rural, other	Health facilities, workplace, other[&]	Health post, rural health facility, local level health centers, municipality level, primary level, sub-metropolitan municipality, urban health care center	March 2021	Health Facility Directors: 21<br>Health Workers: 29<br>Patients: 157	Public
	Thailand^	ND	ND	ND	October 2021	ND	ND
WPR	Laos^	ND	ND	ND	March 2021	Health Facility Directors: 14<br>Health Workers: 30<br>Patients: 38	ND
	Mongolia^	ND	ND	ND	March 2021	ND	ND
	Philippines	ND	ND	ND	October 2021	ND	ND

* AFR – African Region, AMR – Region of the Americas, EMR – Eastern Mediterranean Region, EUR – European Region, SEAR – Southeast Asia Region, WPR – Western Pacific Region

+ - In most cases, the number of health facility Directors also represents the number of health facilities visited

^ - Country has a mature influenza vaccination program for health workers

& - Other facility types included mobile clinics, vaccination centers, and religious organization facilities, such as churches

ND – No data provided.

Table S2. National Immunization Program Maturity Scoring Rubric.

Immunization Program Maturity	
**National Characteristics**	**Scoring/Values**
National Policy for Vaccine –in 2020	Countries were scored by the presence or absence of a national level policy in 2020. Received 1 point if they had a policy for the vaccine. If not, they received a score of 0.
Childhood (DTP[#], MCV[^], PCV[+])	
DTP	
MCV	
PCV	
Adolescent (HPV[′])	
Adult (Influenza)	
Years with Vaccines (0 - 2 points)	Countries were scored by the number of years of experience with each vaccine. Received 2 points if they had 20 or more years using the vaccine of interest. If 10-20 years, they received 1 point and 0 if less than 10 years of experience.
Childhood (DTP, MCV, PCV)	
DTP	
MCV	
PCV	
Adolescent (HPV)	
Adult (Influenza)	
Vaccine Use/Coverage (0 - 2 points)	Countries were scored by the vaccination coverage levels for each vaccine in 2020. Received 2 points if coverage was over 80% in the target population, 1 point if between 50% and 80% and 0 points if less than 50% coverage.
Childhood (DTP, MCV, PCV)	
DTP	
MCV	
PCV	
Adolescent (HPV)	
Adult (Influenza) *	

DTP – Diphtheria, tetanus, pertussis vaccine
^ MCV – Meningococcal vaccine
± PCV – Pneumococcal vaccine
′ HPV – Human papillomavirus vaccine
*Influenza coverage for adults was unavailable, so the availability of influenza vaccines was used. Countries received 1 point if influenza vaccines were available (public and/or private sector) and 0 point if unavailable.

Table S3. COVID-19 Capacity Scoring Rubric.

Score Area	Scoring
I. Evidence Based Decision Making	
Does the country have a NITAG* that contributed to policy decision?	If yes, country received 1 point, if no, 0 points.
II. Waste Management	
Does the country have sufficient treatment and disposal equipment?	If yes, country received 1 point, if no, 0 points.
III. Vaccine Safety System	
Does the country have a functional vaccine safety system in place for COVID-19 vaccine?	If yes, country received 1 point, if no, 0 points.
IV. Surveillance	
Did the country report data from sentinel surveillance on SARS-CoV-2 to WHO during 2021?	If yes, country received 1 point, if no, 0 points.
V. Acceptance and Demand	
Did the country conduct National acceptance and demand assessments for COVID-19 vaccine?	If yes, country received 1 point, if no, 0 points.

* NITAG – National Immunization Technical Advisory Group

Figure S1. COVID-19 vaccine rollout by month, by mature HW influenza vaccination program presence and World Bank income classification; publicly available data (n=60 countries)

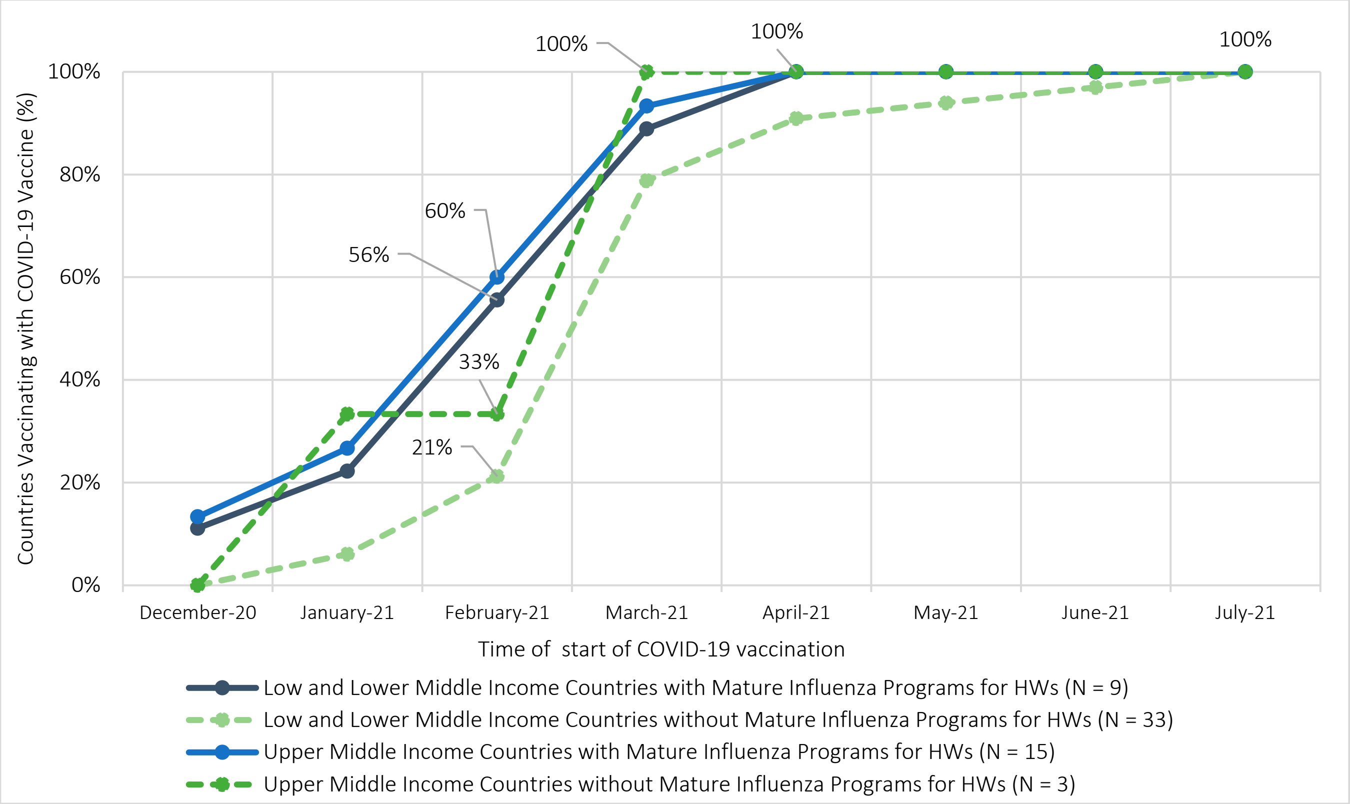

Table S4. General population COVID-19 vaccination coverage 12 months following COVID-19 vaccine deployment, by mature HW influenza vaccination program presence, adjusted for country; publicly available data (n=60 countries)

**All countries**	**Mature Health Worker Influenza Vaccination Program**			
**Timepoint**	Yes	No	Odds Ratio	P-values
**1 month**	2.1 (1.1, 3.9)	1.2 (0.7, 2.2)	1.7 (0.7, 3.9)	0.206
**3 months**	**5.7 (3.5, 9.2)**	**1.8 (1.1, 2.9)**	**3.3 (1.6, 6.6)**	**0.001**
**6 months**	**19.4 (13.3, 27.4)**	**7.6 (5.3, 10.9)**	**2.9 (1.6, 5.3)**	**<0.001**
**9 months**	**38.7 (28.9, 49.5)**	**14.1 (10.2, 19.1)**	**3.8 (2.2, 6.9)**	**<0.001**
**12 months**	**45.7 (35.4, 56.4)**	**24.8 (18.9, 32.0)**	**2.5 (1.5, 4.5)**	**0.001**
**Low + Lower-Middle Income**				
**1 month**	2.0 (0.8, 5.2)	1.3 (0.7, 2.4)	1.6 (0.5, 4.9)	0.427
**3 months**	**5.6 (2.4, 12.4)**	**1.7 (1.0, 2.8)**	**3.4 (1.2, 9.4)**	**0.017**
**6 months**	**20.8 (11.0, 35.8)**	**7.3 (4.9, 10.7)**	**3.4 (1.4, 8.0)**	**0.007**
**9 months**	**43.9 (27.1, 62.2)**	**13.3 (9.3, 18.6)**	**5.1 (2.2, 12.1)**	**<0.001**
**12 months**	**52.4 (34.7, 69.5)**	**21.9 (16.0, 29.2)**	**3.9 (1.7, 9.1)**	**0.001**
**Upper-Middle Income**				
**1 months**	2.2 (1.0, 4.6)	1.2 (0.2, 6.8)	1.8 (0.3, 11.5)	0.550
**3 months**	5.8 (3.2, 10.0)	2.8 (0.7, 10.4)	2.1 (0.5, 9.3)	0.323
**6 months**	19.0 (12.5, 27.7)	13.4 (4.8, 32.1)	1.5 (0.4, 5.1)	0.506
**9 months**	36.1 (26.1, 47.5)	25.3 (10.5, 49.5)	1.7 (0.5, 5.3)	0.386
**12 months**	42.3 (31.7, 53.7)	58.4 (34.9, 78.7)	0.5 (0.2, 1.5)	0.231

Table S5. Reported health worker COVID-19 vaccination coverage, cPIE responses, by mature HW influenza vaccination program presence; (n=10 countries; 262 health workers=)

	Mature Health Worker Influenza Vaccination Program			
	Yes (n=118)	No (n=144)	All Health Workers	p-value
**Across All HWs, n, (%)**				
At least 1 dose	113, (98)	143, (99)	256, (99)	0.136
At least 1 booster dose#	59, (92)	78, (69)	162, (77)	0.502
**At Least 1 Dose, by Geographic Region, n, (%)**				
African	13, (93) <	87, (99)	100, (98)	0.640
Eastern Mediterranean >	21, (100)	27 (100)	48, (100)	Na
European	50, (100)	ND	50, (100)	Na
Southeast Asia^	ND	29, (100)	29, (100)	Na
Western Pacific*	29, (97)	ND	29, (97)	Na
**Booster Dose Vaccination, by Geographic Region, n, (%)**				
African &	ND	54, (62)	54, (62)	Na
Eastern Mediterranean>	18, (86)	24, (92)	42, (89)	0.800
European	41, (95)	ND	41, (95)	Na
Southeast Asia	ND	ND	ND	Na
Western Pacific	ND	ND	ND	Na
**At Least 1 Dose, by World Bank Income, n, (%)**				
Low Income$	ND	16, (100)	16, (100)	Na
Lower Middle Income	87, (98)	127, (99)	214, (99)	0.750
Upper Middle Income	26, (100)	ND	26, (100)	Na
**Booster Dose Vaccination, by World Bank Income, n, (%)**				
Low Income$	ND	8, (50)	8, (50)	Na
Lower Middle Income+	41, (91)	70, (72)	111, (78)	0.011
Upper Middle Income	18, (95)	ND	18, (95)	Na

= - Denominators for each category and grouping differ based upon data availability
# - Data from seven countries – Albania, Armenia, Ghana, Lebanon, Mali, Tajikistan, and Tunisia
< - Data from one country – Cote d'Ivoire
> - Data from two countries – Lebanon and Tunisia
^ - Data from one country – Nepal
* - Data from one country – Laos
& - Data from two countries– Ghana and Mali
$ - Data from one country – Mali
+ - Data from four countries – Ghana, Lebanon, Tajikistan, and Tunisia
